# Management of patients with dysthyroid optic neuropathy treated with intravenous corticosteroids and/or orbital decompression surgery

**DOI:** 10.1007/s00417-022-05732-4

**Published:** 2022-06-22

**Authors:** Sandra Rezar-Dreindl, Andrea Papp, Arnulf Baumann, Thomas Neumayer, Katharina Eibenberger, Eva Stifter, Ursula Schmidt-Erfurth

**Affiliations:** 1 Department of Ophthalmology and Optometry, Medical University of Vienna, Waehringer Guertel 18-20, A-1090 Vienna, Austria; 2 Department of Oral and Maxillofacial Surgery, Medical University of Vienna, Vienna, Austria

**Keywords:** Thyroid eye disease, Dysthyroid optic neuropathy, Intravenous corticosteroids, Orbital decompression surgery

## Abstract

**Purpose:**

To assess the characteristics and long-term outcomes of adult patients with dysthyroid optic neuropathy (DON) who underwent orbital decompression surgery and/or received intravenous (IV) methylprednisolone.

**Methods:**

Retrospective chart review of 98 eyes of 49 patients who were diagnosed and treated with bilateral DON between 2007 and 2018 at the Department of Ophthalmology and Optometry and Oral and Maxillofacial Surgery of the Medical University of Vienna.

**Results:**

The mean follow-up period was 4.1 ± 2.7 years. The most common presenting symptoms were eyelid and periorbital swelling (45%) representing active inflammation. Upgaze restriction was the most common clinical finding (73%). At time of diagnosis, the mean clinical activity score was 4 ± 1/4 ± 1 (right/left eye, respectively). Sixty-three percent (31/49) of the patients were treated both with IV methylprednisolone and underwent orbital decompression surgery, 22% (11/49) were treated with IV methylprednisolone alone and 14% (7/49) underwent surgical decompression only. Seventy-one percent (30/42) of the patients underwent 3-wall decompression. The mean reduction of proptosis in patients treated with both IV methylprednisolone and orbital decompression surgery was 4/5 mm. Mean of reduction in proptosis in patients receiving IV methylprednisolone only was 1/0 mm and in patients with surgical decompression only was 5/5 mm. Mean VA was 0.1 ± 0.5/0.1 ± 0.5 logMAR at baseline and 0.05 ± 0.7/0.05 ± 0.7 at final follow-up. In 92% (45/49), VA was preserved or improved at final follow-up.

**Conclusions:**

The majority of patients with DON were treated both with IV corticosteroids and 3-wall decompression surgery. Vision could be successfully preserved in most cases and reduction of proptosis was achieved, especially after orbital decompression surgery.

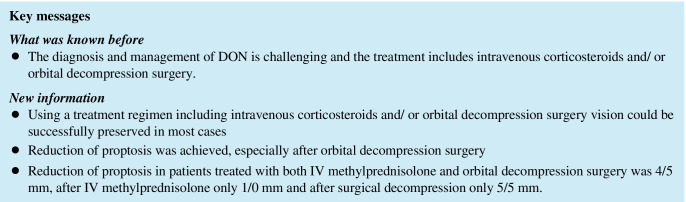

## Introduction

Thyroid eye disease (TED), Graves’ orbitopathy or thyroid-associated orbitopathy, is the main extrathyroidal manifestation of Graves’ disease and is strongly associated with a reduced quality of life and socioeconomic status. In up to 50% of patients with Graves’ disease, TED can become clinically relevant [1–5]. Early diagnosis and a distinct management at a specialised centre including endocrinologists, ophthalmologists and orbit surgeons is important to prevent a potentially sight-threatening course of disease and the development of dysthyroid optic neuropathy (DON). DON accounts for about 3–7% of all cases with TED [6, 7]. The diagnosis of DON can be challenging as the visual acuity (VA) often remains unimpaired during a long period of time [8]. In addition, some patients can present without severe proptosis and signs of inflammation [9].

Many patients with TED suffer from only mild to moderate symptoms. In those cases, intake of selenium, modification of lifestyle factors (smoking cessation) and optimal management of hyperthyroidism can prevent progression of disease [10, 11]. In patients who present with moderate-to-severe or active TED treatment with intravenous glucocorticoids with or without mycophenolate sodium is initiated as first-line treatment. Second-line therapies may include a second cycle of glucocorticoids, cyclosporine or azathioprine, orbital radiotherapy, teprotumumab, rituximab and tocilizumab. For patients who develop sight-threatening disease and DON, the first-line treatment is high-dose intravenous methylprednisolone. If there is no or poor response, orbital decompression surgery should be done to prevent permanent vision loss [12]. Glucocorticoids applied intravenously are often used as a first-line therapy to treat patients with DON. However, high doses are needed to achieve visual recovery and to stop further disease progression. Recurrences are likely once the treatment with glucocorticoids is stopped and side effects need to be carefully considered [13]. However, the level of evidence is low for the treatment of patients with DON [12].

Surgical decompression is the other pillar for TED. The goal is to reduce orbital tissue pressure to restore vision and function of the extraocular muscles and improve eyelid closure and proptosis. There have been described various decompression techniques since the first report by Dollinger in 1911 and also indication for surgery has changed over time. Classically, surgery was reserved for DON and extreme corneal exposure. Currently, indications are broad (cosmetic/ function) and technique is often graded and individualised [14]. The different surgical techniques are associated with variable amounts of proptosis reduction and complication rates. However, the optimal timing and indication for surgical intervention remain controversial [7, 15, 16]. To date, no consensus exists regarding the treatment and managing strategy for patients who develop DON. This study aimed to evaluate the characteristics and long-term outcomes of patients with DON using a managing strategy with IV corticosteroids and/or orbital decompression surgery.

## Methods

In this retrospective analysis, patients with DON were included, who were treated at the Department of Ophthalmology and Optometry and the Department of Oral and Maxillofacial Surgery of the Medical University of Vienna between 2007 and 2018. Only patients with bilateral DON were included, if a minimum follow-up of 1 year after initial presentation was recorded in the electronic charts. Patients receiving orbital radiotherapy for DON were excluded from the analysis. The patients were evaluated regarding their characteristics, course of disease and management strategies. The study was approved by the local ethics committee of the Medical University of Vienna. The study design adheres to the tenets of the Declaration of Helsinki.

DON was defined according to EUGOGO as patients with TED and having at least two of the following features not attributable to any other cause: impaired visual activity (VA; subjectively and/or objectively), reproducible defects on automated perimetry, impaired colour vision, muscle index measured via standardised ultrasound of ≥ 7 or documented compression of the optic nerve on magnet resonance tomography (MRT) or computer tomography (CT) imaging, optic disc swelling or elevated intraocular pressure (IOP) of ≥ 25 mmHg [17]. Visual acuity was evaluated objectively using Snellen visual acuity charts or subjectively by report of the patient. Farnsworth D15 test was done to detect anomalies of colour vision. Muscle index was calculated using orbital sonography. Ossoinig muscle index was calculated which summed-up extraocular muscle thickness measurements divided by the number of the summed-up muscles. An index < 5 is graded as normal, 4.5–5.5 represents mild ophthalmopathy, 5.5–6.5 moderate ophthalmopathy and an index > 6.5 is severe ophthalmopathy [18]. Radiological signs in MRT or CT include optic nerve compression at the orbital apex by enlarged extraocular muscles (apical crowding) or optic nerve stretching [19].

Medical records of all patients were analysed at first presentation and at subsequent visits. The ophthalmic parameters such as VA, anomalies of the anterior and posterior part of the eye, eye pressure and their changes during the treatment by IVMP or surgical orbital decompression therapy were analysed.

Management of the patients was done by interdisciplinary consultation of the Department of Ophthalmology, Endocrinology and Oral-Maxillofacial Surgery. Treatment of inflammatory orbitopathy was first done according to Kahaly et al. [20] and later according to EUGOGO [17, 21]. The CAS < 3 should be confirmed before surgery, to discriminate between active and quiescent stages of the disease. The surgical decompression therapy was a 3-wall or a 2-wall decompression procedure. The indication for a 3-wall decompression was a more prominent existing exophthalmos and enlarged (thickened) eye muscle in the CT/MRT scan. In the other cases, a 2-wall decompression was done. Therefore, the surgical procedure depended on the anatomical findings (CT) and the clinical ophthalmic parameters. If the visual impairment was more severe, a 3-wall decompression was done. All decompression surgeries were performed by one surgeon (AB).

### Surgical technique

The surgical technique consisted of trepanation and resection of the orbital floor and medial wall (2-wall decompression) under general anaesthesia and orotracheal intubation. The approach was done by a transconjunctival approach in the inferior fornix without a lateral canthotomy. The dissection continued in the preseptal plane down to inferior rim of the orbit, where the periosteum was incised. The orbital floor was exposed and medial of the infraorbital nerve the trepanation is started behind the orbital rim. Further bone resection of the orbital floor and medial wall in the dorsal orbit was preceded under surgical navigation. In cases where bone resection was necessary till the level of the ethmoidal foramina, an additional transcaruncular incision was used. Finally, the periorbit was incised in the medial and caudal part to allow fat protrusion. Additionally, fat was removed from the medial lower compartment.

In a 3-wall decompression, the procedure starts as described for the 2-wall decompression. Additionally, a “C” formed lateral osteotomy of the lateral orbital rim was done through the zygoma corpus. The approach for this osteotomy is a lateral eye brow incision or upper eyelid blepharoplasty incision and a buccal oral incision. The caudal part of the “C” osteotomy is done during the 2-wall decompression. Starting lateral of the infraorbital nerve, the orbital rim is cut with piezo surgery down to the maxilla. The caudal orbital floor is separated behind the orbital rim by piezo surgery into the lateral orbit. The upper part of the “C” osteotomy is then done at the sutura zygomaticofrontalis by removing a bone edge. The eye is protected by a dura spatula. The bone of the lateral orbital wall is cut from the suture to the caudal anterior lateral part of the orbit by piezo surgery and with the chisel. Subperiosteal dissection of the lateral wall is done to the caudal anterior lateral part of the orbit under protection of the periorbita. The bone cut is connected with the lower part of the orbit. A buccal incision is necessary to complete the “C” osteotomy. Below the lateral infraorbital nerve, the bone is cut with a saw directed towards the connection of the caudal lateral orbital wall and the zygomatic arch. For complete mobilisation, a chisel is used. The “C” lateral orbit ring can then be moved anteriorly and laterally to enlarge the orbit. The segment is fixed by miniplates at the zygomatic suture and lateral of the infraorbital nerve. The bone of the lateral wall can further reduced as described by Goldberg et al. [22].

### Surgical navigation device

A preoperative computed tomography (CT) of the facial skeleton in a multi-detector spiral technique, without contrast agent, was performed with 0.8-mm slice thickness in axial and 3-D reconstruction with 1.5-mm thickness for the coronal reconstruction. These CT data were used for preoperative diagnosis. The Digital Imaging and Communications in Medicine (DICOM) data were transferred to the navigation station. The Medtronic Treon Navigation station (Medtronic Inc, Minneapolis MN, USA) was used till 2012 for intraoperative navitation. From 2012, the Brainlab Curve™ single display navigation station (Brainlab AG, Munich Germany) was used for intraoperative navigation. Registration of the radiological imaging data with the patient was then conducted. In the Medtronic system, the face was registered by anatomical points. In the Brainlab system, a surface matching was used based on laser surface scanning. The consistency of the calibration of the patient was checked in both systems by identification of several anatomic points in the orbital region.

The surgical navigation system allowed with the tip of an instrument (pointer) the three-dimensional location in the operating field at any time. The saw cuts in the infraorbital region can be preceded with the navigation system fixed on the instrument. The decision to use the navigation system depends on the specific conditions of the patient and the surgeon’s experience.

### Statistical analysis

Statistical analysis was performed using descriptive statistics for all variables and data frequency. The aim of the analysis was to evaluate the management of patients with DON treated with orbital decompression surgery and/or IVMP. Baseline variables were analysed for all the patients with DON. The change of VA, orthoptic parameter and IOP over time was determined. Information regarding treatment was described.

## Results

Forty-nine patients were included in this retrospective study. Forty-nine patients (12 male and 37 female) with bilateral DON (98 eyes) were treated with orbital decompression surgery and/or IVMP. The mean age of the patients was 52 ± 14 years (median 51; range 29–82 years) at initial presentation. The mean follow-up period was 4.1 ± 2.7 years (median 3.5; range 1–12.2 years) in total.

Duration of thyroid disease before diagnosis of DON was 38 ± 48 months (median: 24 months; range from 0 to 216 months). Eighty-six percent of the patients had DON at initial presentation (42/49), and in 14% (7/49), DON developed during the follow-up after a mean time of 3 ± 6 months. Eighty-eight percent (43/49) had diagnosis of Basedow hyperthyroidism. Twelve percent (6/49) had diagnosis of Hashimoto thyroiditis. The documented thyroid specific medication was thiamazole in 47% (23/49), prothiucil in 8% (4/49) and levothyroxin-natrium in 14% (7/49). Three patients (10%) had no therapy at the time of presentation, and in 20% (10/49), information about the current treatment was not available.

At initial presentation, patient’s blood test revealed active hyperthyreosis in 61% (30/49). Thyrotropin receptor autoantibodies were elevated in 69% (34/49) of the patients. Sixteen percent (8/49) of the patients with DON had a history of thyroidectomy before initial presentation. In 5 of the 8 patients, thyroidectomy was performed within 1 year before diagnosis of DON (mean 5 ± 4 months). In the remaining 3 patients, who showed a long disease duration, thyroidectomy was performed more than 1 year before diagnosis of DON (mean 9 ± 3 years). Three out of the 5 patients with thyroidectomy within 1 year did not receive oral prednisolone after thyroidectomy. Eight percent (4/49) of the patients had history of radioactive iodine with a mean time of 6 ± 1 months before diagnosis of DON. All four cases of DON following radioactive iodine did not receive oral prednisolone therapy.

Twenty-two percent (11/49) of the patients had concomitant diabetes mellitus, 16% (8/49) hypertension, 6% (3/49) had cardiomyopathy and 6% (3/49) hypercholesterinaemia. Sixty-three percent (31/49) of patients were smokers or ex-smokers and only 18% (9/49) never smoked (missing 18%, 9/49).

### Clinical symptoms and CAS

Thirty-nine percent (19/49) of the patients reported one symptom at initial presentation and 59% (29/49) reported more than one symptom. The most common presenting symptom which was reported by the patients was eyelid and periorbital swelling (49%, 24/49). Table 1 shows the clinical symptoms reported by the patients at initial presentation. The mean documented CAS at initial presentation was 4 ± 1/4 ± 1 (median: 4/4; right/left eye). Fourteen percent (7/49) of the patients showed minimal inflammation with a documented CAS of 0–2 at initial clinical presentation.

**Table 1:** Clinical symptoms reported by the patients with DON at initial presentation (*n* = 49)

Clinical symptoms reported by the patients	Percentage (number)
Eyelid and periorbital swelling	49 (24)
Diplopia	43 (21)
Reduced or blurred vision or colour vision	35 (17)
Symptoms comparable to sicca syndrome	35 (17)
Pain	8 (4)
Noticed visual field defects	2 (1)
No symptoms	2 (1)

Mean VA at initial presentation was 0.1 ± 0.5/0.1 ± 0.5 logMAR (median: 0.05/0.05; range − 0.1–0.4/ − 0.1–1.6 logMAR). At initial presentation, clinical examination showed upgaze restriction as the most common finding in 73% (36/49) of patients. Mean proptosis was 23 ± 3 mm/23.5 ± 4 mm in the right/left eye (median: 23/23.5 mm; ranges 17–31 mm). Width of the vertical palpebral fissure was 11 ± 2.5/11 ± 2.7 (median: 11/11; range: 6–19/5–17). Measurement of the superonasal muscle index (SNI) via ultrasound was 7.1 ± 0.4/7.1 ± 0.5 (median: 7.0/7.0; range: 6.3–8.1/6.2–8.39). Sixty-one percent (30/49) had documented visual field defects with concentric narrowing. Table 2 shows information about ocular motility and proptosis at initial presentation of the patients with DON.

**Table 2:** Ocular motility and proptosis in patients with DON at initial presentation (*n* = 49)

Restriction of ocular motility	Percentage (number)
Upgaze restriction	73 (36)
Restriction of upgaze and abduction	61 (30)
Restriction of upgaze and adduction	20 (20)
Upgaze restriction only	8 (4)
Restriction of abduction only	8 (4)
Proptosis in mm (right/left)	Percentage (number)
Proptosis 23–25 mm	43/35 (21/17)
Proptosis 26–30 mm	18/22 (9/11)
Proptosis > 31 mm	2/4 (1/2)

### Treatment and outcome of patients with DON

Patients were treated with either IVMP and/or orbital decompression surgery after diagnosis of DON. After the diagnosis of DON, 86% (42/49) of the patients were immediately treated with IVMP. In 76% (32/42) of the patients, IVMP 500 mg/week for 6 weeks followed by 250 mg/week for 6 weeks was initiated. Twenty-four percent (10/42) of the patients received high-dose methylprednisolone for 3 consecutive days (500 mg each day). Five hundred milligrams of IVMP/week for 6 weeks followed by 250 mg/week for 6 weeks was given in 73% of patients who received IVMP only (*n* = 11) and high-dose MP for 3 days in 27% with a mean cumulative IVMP dose of 3.7 ± 1.3 g. Five hundred milligrams of IVMP/week for 6 weeks followed by 250 mg/week for 6 weeks was given in 74% of patients who received IVMP and decompression surgery (*n* = 31) and high-dose MP for 3 days in 26% with a mean cumulative IVMP dose of 3.4 ± 1.4 g.

In 22% (11/49), no surgical intervention was performed after IV steroid treatment. Seven of these patients showed good overall improvement after IVMP and the visual field defects recovered. Two patients declined surgical intervention and the other 2 patients had severe risk factors, which were a contraindication for general anaesthesia.

Fourteen percent (7/49) of the patients did not receive IVMP treatment and were referred to orbital decompression only. Five of the 7 patients had pre-existing contraindications for steroid treatment (gastrointestinal ulceration, septic arthritis). In two patients, immediate orbital decompression without previous steroid treatment was done due to high exophthalmus. Sixty-three percent (31/49) of the patients underwent orbital decompression surgery after IVMP.

At the last follow-up, mean proptosis of the patients was 21 ± 4/20 ± 4 mm in the right/left eye (median: 20/20 mm). The mean change compared to initial presentation in all patients was 2/4 mm. Patients treated with IVMP and orbital decompression surgery showed a mean change in proptosis of 4/5 mm (right/left) (*n* = 31). The mean change in proptosis in patients receiving IVMP was 0/1 mm (*n* = 11) and in patients with surgical decompression only 5/5 mm (*n* = 7). Out of the 38 patients with orbital decompression surgery, 79% (30/38) had a 3-wall decompression surgery. In patients receiving a 3-wall decompression, change in proptosis was from 24/24 to 19/19 mm at final follow-up (*n* = 30). A 2-wall decompression was done in 21% (8/38). In patients with 2-wall decompression, a change in proptosis was from 22/23 to 20/20 mm at the final follow-up (*n* = 8).

Mean VA at the last follow-up was 0.1 ± 0.7/0.1 ± 0.7 logMAR (median: 0.0/0.0; range: 1.3 to − 0.1). Four percent (2/49) of the patients had final VA of ≥ 0.5 logMAR in one eye. One patient had VA of > 0.3 logMAR in one eye. Ninety-four percent (46/49) had final VA of 0.3 or better in both eyes. In 92% (45/49), VA was preserved or improved at final follow-up in at least one eye of each patient. The VA at the final follow-up after combined IVMP and orbital decompression surgery was 0.05 ± 0.5/0.1 ± 0.7, 0.05 ± 1.0/0.05 ± 1.0 after IVMP only and 0.0 ± 0.7/0.1 ± 0.5 after orbital decompression surgery only. Tables 3 and 4 summarise the mean proptosis and VA of the patients with DON at initial presentation and last follow-up.

**Table 3:** Change of proptosis in patients with DON at initial presentation and last follow-up (*n* = 49)

Proptosis in mm	Initial presentation (right/left)	Last follow-up (right/left)	*p* value
	Mean ± standard deviation (median)	Mean ± standard deviation (median)	
All patients	23 ± 3/24 ± 4 (23/24)	20 ± 4/20 ± 4 (20/20)	< 0.01
IV MP and surgery (*n* = 31)	23 ± 3/24 ± 4 (23/23)	20 ± 3/19 ± 3 (20/19)	< 0.01
IV MP only (*n* = 11)	23 ± 4/24 ± 4 (23/25)	22 ± 5/22 ± 4 (19/20)	0.8
Surgery only (*n* = 7)	24 ± 2/24 ± 3 (23/23)	19 ± 2/20 ± 2 (23/23)	< 0.01

**Table 4:** Change of visual acuity in patients with DON at initial presentation and last follow-up (*n* = 49)

Visual acuity (logMAR)	Initial presentation (right/left)	Last follow-up (right/left)	*p* value
	Mean ± standard deviation (median)	Mean ± standard deviation (median)	
All patients	0.05 ± 0.5/0.1 ± 0.7 (0.0/0.05)	0.05 ± 0.5/0.1 ± 0.3 (0.0/0.0)	0.5
IV MP and surgery (*n* = 31)	0.05 ± 0.5/0.05 ± 0.7 (0.0/0.0)	0.1 ± 0.4/0.1 ± 0.4 (0.0/0.0)	0.2
IV MP only (*n* = 11)	0.05 ± 0.5/0.1 ± 0.5 (0.1/0.0)	0.05 ± 1.0/0.05 ± 1.0 (0.0/0.0)	0.3
Surgery only (*n* = 7)	0.1 ± 0.7/0.1 ± 0.1 (0.05/0.05)	0.0 ± 1.0/0.1 ± 0.5 (0.05/0.05)	0.4

One patient had pre-existing amblyopia, one had diabetic retinopathy and in one patient neovascular age-related macular degeneration was present limiting VA results. Six percent (3/49) experienced visual loss (in one or both eyes) despite surgical intervention. The mean VA outcome in those patients was 0.1 ± 0.5/0.4 ± 0.7 (right/left eye).

Width of the vertical palpebral fissure at the final follow-up was 9 ± 3/9 ± 3 (range 4–16/2–16). Twenty percent (10/49) of the patients received thyroidectomy 0.7 ± 0.5 months after initial diagnosis of DON. Highest mean intraocular pressure documented during the follow-up period was 23 ± 5/23 ± 4 mmHg. Ocular hypertension, defined as intraocular pressure of ≥ 25 mmHg, was found in 24% (12/49) of the patients. Patients with ocular hypertension were all treated with topical IOP medication. Twenty-five percent (3/12) of the patients with ocular hypertension were treated with topical IOP medication only, and 42% (5/12) received combined therapy of topical and systemic IOP medication.

### Diplopia and rehabilitative surgery

In patients following decompression surgery, 18% (7/38) had diplopia and needed prisma therapy at final follow-up and 11% (4/38) had diplopia only in up- or downgaze. Five patients (13%) showed no restriction of motility at final follow-up. Fifty-eight percent (22/38) showed minor abduction and/or upgaze restriction, and 11% (4/38) patients had minor abduction, adduction, upgaze restriction and downgaze restriction. One patient (3%) had strabismus convergens and hypotropia. In 16% (6/38), upgaze was considerably restricted at final follow-up.

Twenty-seven percent (3/11) of patients without surgical decompression showed normal motility, 55% (6/11) had minor upgaze and/or abduction restriction and in one patient (9%) strabismus convergens with hypotropia existed at final follow-up. Also, one patient had diplopia in upgaze at final follow-up.

In 53% (26/49), rehabilitative surgery including eyelid and/or strabismus surgery was necessary. In patients following orbital decompression surgery, 29% (11/38) had no rehabilitative surgery, 55% (21/38) had strabismus and lid surgery and in 16% (6/38) only lid surgery was performed. The rehabilitative surgery of the 26 patients was performed after a mean of 1.5 ± 9 months after the decompression surgery.

In the group of patients without surgical decompression, 73% (8/11) had no rehabilitative surgery, 9% (1/11) needed strabismus and lid surgery and in 9% (1/11) only lid surgery was performed.

## Discussion

In patients with DON, a multidisciplinary approach is necessary to avoid severe and irreversible vision loss [3]. Rapid treatment initiation is important and the treatment strategies mainly include glucocorticoids and surgical decompression. Still, little evidence exists about the dose of glucocorticoids and there is the need to determine the best therapy and the timing for the treatment of patients with DON.

This study presents an evaluation of the clinical characteristics of sight-threatening DON and displays long-term outcomes of the treatment strategies including IVMP and/or orbital decompression surgery.

Patients with severe TED should be managed in a specialised centre or combined thyroid eye clinic. Many patients are referred to a specialised centre too late and this can lead to a delay in treatment and visual loss [21]. The majority of our patients were diagnosed with DON at initial presentation. Only 16% presented with severe TED developed DON during the follow-up. The initial characteristics of patients with DON are in agreement with other studies [23]. Increased age and vascular disease are risk factors for the development of DON. These patients have a worse outcome after treatment [6, 24]. In our study, only bilateral cases were included and the most common presenting symptoms were lid and periocular swelling, presenting an active inflammation. Also, mean CAS was 4/4 in our study patients representing disease activity. On clinical examination, upgaze restriction was the main finding in 73%. Upgaze restriction could be an important clinical finding which should lead to a closer monitoring of patients with TED to prevent worsening and ensure an early diagnosis of DON. Also > 50% of our patients with DON had a present hyperthyreosis with high thyrotropin receptor autoantibody levels. Increased levels of autoantibodies were shown to correlate with a more severe disease and could serve as a prognostic marker [25]. Knowledge about the status of the thyroid is important as risk of progression of TED or development of DON. Also a good interdisciplinary collaboration is necessary of patients with DON as euthyroidism should be promptly reached and stably maintained.

Visual acuity of our patients with DON showed a wide range from − 0.1 to 1.6 logMAR at initial presentation. This again shows the variability of presenting features and heterogeneous disease patterns leading to a personalised treatment regimen. Furthermore, diagnosis of DON can be difficult when VA is normal, misleading to a delay of treatment initiation. Also, the degree of proptosis in our patients varied between 17 and 31 mm at initial presentation. For diagnosis of DON, it is important to be aware of the fact that due to a tight orbit the optic nerve can be compressed without clinical significant proptosis. Detecting subtle evidence of DON is crucial for early diagnosis and adequate treatment.

Our data showed that in the majority of patients 63%, treatment was started with IVMP followed by surgical intervention. Twenty-two percent of the patients received only IVMP. The reasons why patients were not treated further with orbital decompression were good and rapid improvement after IVMP. Four of these patients declined a surgical intervention or there were severe risk factors. On the other side, 7 patients had primary surgery. Orbital decompression was done because of contraindications for glucocorticoids, massive proptosis or low inflammation and rather fibrotic residuum causing compression.

In 2016, the EUGOGO published treatment guidelines for patients with TED, which recommended treatment with high doses of IV glucocorticoids for DON. If there is a good response to the treatment resulting in an inactivity of disease, rehabilitative surgery can be done if needed [21]. An urgent orbital decompression should be performed if poor/no response is noticed within 2 weeks after treatment initiation with IVMP. If DON has improved after 2 weeks, IVMP should be continued. However, due to the complexity of the disease with different presenting characteristics, the treatment regimen may vary depending on clinical parameters. One has to consider co-morbidities and potential side effects of glucocorticoids. Our patients received either a treatment regimen with 500 mg/week for 6 weeks followed by 250 mg/week for another 6 weeks (Kahaly) or received 500 mg MP/day for 3 consecutive days. Within our analysis, no serious side effects occurred using the IVMP regimen. However, only 11% of these patients received the high-dose glucocorticoids for 3 days. This could be attributed to the fact that in our patients IVMP was given before the recommendations for high-dose glucocorticoids in 2016 (EUGOGO). In the 2008 consensus statement, high-dose IVMP administered in pulses is recommended for patients with DON (Kahaly), and according to Hart Frantzco RH et al., improvement of optic nerve function may be expected after high-dose IV steroids (1.5 g for 3 days). However, this was a prospective, non-controlled case series including 18 patients [1]. Further studies including more patients with DON and directly comparing the different steroid treatment regimens in prospective, randomised studies are necessary to determine the optimal steroid regimen for this group of patients.

In our study, 31 patients (63%) were referred to surgical decompression after prior IVMP. One randomised, controlled trial evaluated surgery versus steroids. Six were treated with surgical decompression and 9 patients received IVMP. In this study, immediate surgery was shown to not result in a better outcome when compared to MP. The treatment regimen used included MP 1 g daily for 3 consecutive days which was repeated after 1 week. This was followed by oral prednisone for 4 months [26]. Another study also evaluated the effectiveness of IVMP in patients with DON. IVMP was given daily for 3 days (500 or 1000 mg) which was repeated the following week. The steroids were tapered off with oral or IV steroids. Forty-three percent of eyes showed complete visual recovery and surgical intervention was not necessary, which is lower when compared to our patients [15]. Cumulative steroid dose was lower in our group of patients. In our evaluation, most patients received 500 mg IVMP weekly for 6 weeks followed by 250 mg for 6 weeks. Later, patients were treated with higher doses including 500 mg IVMP for 3 consecutive days. It is possible that higher doses of steroids may improve the rate of patients without the need of surgical decompression. However, in the study by Wakelkamp et al., all patients in whom therapy failed were switched to the other treatment arm and visual acuity improved in almost all patients [26]. The course and outcome of patients with DON is not always predictable. Due to the variable course of disease, individual treatment regimen is necessary. Evaluating the individual risk profile in respect to high doses of steroids is of special importance in this group of patients.

The goal of orbital decompression surgery is to reduce orbital tissue pressure, to restore vision and function of the extraocular muscles, and improve eyelid closure and proptosis. Reduction of exophthalmus varies in previous studies and is reported to be 3–5 mm on average after surgery [27, 28]. Both 2- and 3-wall decompression surgeries were shown to be successful for the treatment of those patients. In our study, most patients were treated with 3-wall decompression. We found in our patients that functional parameter could be preserved. There was also a low rate of postoperative complications such as haemorrhage, infection or further visual decline. Due to the wide variety of surgical techniques and approaches, reduction of exophthalmos differs and therefore the technique has to be adapted for each patient. This makes a comparison of the studies more difficult, because there are differences in each patient. Studies comparing 3-wall and 2-wall techniques reported an increased reduction among the patients who received 3-wall decompression surgery which is in accordance with our study showing a greater reduction after decompression of the medial and lateral wall and the orbital floor [29–31]. The 3-wall decompression was more effective because of the enlargement of the orbit. However, surgery time is longer and resulted in a larger recovery time due to the buccal incision and “C” osteotomy. The anterior and lateral movement of the orbital rim had also an aesthetic improvement of the eyelid crease and can reduce the risk of temporal hollowing. The muscle has not been separated [32].

Looking at the long-term functional outcome, 77% of patients were reported to have normal VA after surgical intervention [27]. In our analysis, 94% of patients had VA of better than 0.3 logMAR at last follow-up under treatment with IVMP and/or orbital decompression surgery. There are only a few randomised trials comparing different treatment strategies in patients with DON. In a study comparing IVMP with orbital decompression, the immediate surgery does not result in a better outcome. Reduction of proptosis was 1 mm in this study after 26 weeks. Based on these findings, the author concluded that IVMP appears to be the first-choice therapy [26]. Among our 11 patients treated with IVMP alone, a reduction of 0/1 mm was recorded. Furthermore, the 6 surgical cases in this study received a 3-wall decompression and proptosis reduced from 24 ± 3 to 22 ± 4 mm at 26 weeks and to 19 ± 2 mm after 52 weeks [26]. This is similar to our patients receiving 3-wall decompression surgery with a reduction of 24/24 to 19/19 mm. So an individual treatment approach including both steroids (first-line) and decompression surgery is necessary in patients with DON based on the presenting features and co-morbidities. Also, special attention should be paid on patients with risk of DON but without significant exophthalmus. In those patients, rapid surgical intervention may be more necessary in order to ease muscle restrictions.

Beside treatment strategies including glucocorticoids and surgical options, biologic agents are currently under evaluation for the treatment of patients with thyroid eye disease. Recently, many reports showed effectiveness of biological agents for the treatment of TED either as monotherapy or in combination with corticosteroids [33, 34]. These new treatment modalities show promising results and the role for the treatment of vision-threatening DON needs to be further investigated.

Limitations that need to be mentioned include the varying follow-up of the patients and the retrospective nature. However, with this investigation, the different presenting features of patients with DON could be shown. They have to be considered in patients with DON in a multidisciplinary team approach. Exact evaluation of the different features is important to identify patients with a high risk for development of DON which have to be monitored closely. Early diagnosis is essential in order to preserve visual function. Depending on the clinical improvement following treatment initiation with IVMP, surgical intervention has to be done. The majority of patients with DON were treated with combined IVMP and 3-wall orbital decompression surgery. Vision could be preserved in the majority of patients with improvement of exophthalmometry. The greatest reduction of proptosis was found in patients following surgical intervention. Therefore, individual planning of treatment and clinical management in accordance to activity and severity of TED including both steroids and decompression surgery is required in patients with diagnosis of DON.
